# Prediction of time to prosthesis implantation as a function of joint anatomy in patients with developmental dysplasia of the hip

**DOI:** 10.1186/s13018-019-1511-4

**Published:** 2019-12-30

**Authors:** Michael Müller, Anasthasia Rakow, Georgi I. Wassilew, Tobias Winkler, Carsten Perka

**Affiliations:** 1 Department of Orthopedics, Center for Musculoskeletal Surgery, Charité - University Medicine Berlin, Augustenburger Platz 1, 13353 Berlin, Germany; 2 Department of Orthopedics and Orthopedic Surgery, University Medicine Greifswald, Greifswald, Germany

**Keywords:** Developmental dysplasia of the hip, Total hip arthroplasty, Prediction of time, Hip joint anatomy, Timing of primary THA, Hip pain

## Abstract

**Background:**

Developmental dysplasia of the hip (DDH) can lead to pain and premature secondary osteoarthritis at an early stage. Joint-preserving osteotomy is an established solution to this problem. In contrast, a conservative approach would result in pain persistence, ultimately raising the patients question for a possible date of expected prosthesis implantation.

The aim of the study was to identify the relationship between the dysplastic hip anatomy and the time of prosthesis implantation in order to enable prognostic predictions in younger patients with symptomatic DDH.

**Materials and methods:**

Data from 129 hips who received THA due to secondary DDH osteoarthritis were evaluated. The preoperative hip anatomy was evaluated for AI and LCE angle. Multiple linear regression analyses were then used to correlate the influence of these parameters with the patient’s age at the time of surgery. In addition, a graphical relationship was derived by the method of power least squares curve fitting with second-degree polynomials.

**Results:**

The mean age for THA was 54.3 ± 11 years. The time of surgery correlated significantly with LCE (0.37) and AI (− 0.3) (*p* < 0.001). The mean age of patients with LCE angle ≤ 10° was 41.9 ± 14.0 years, for LCE 11–20° 52.7 ± 9.5 years, and for LCE 21–30° 57.0 ± 10.3 years. The following formula could then be determined for the calculation of the potential patient age at the time of THA as a function of LCE angle: age pTHA = 40.2 + 0.8 × LCE angle − 0.01 × (LCE angle)^2^.

**Conclusion:**

A significant correlation between the extent of dysplasia and the time of prosthesis implantation was identified. In particular, the LCE and the AI correlated strongly with the time of implantation. The more dysplastic the angles were, the sooner the THA was necessary. Using the calculations presented in this study, the probable age of prosthesis implantation can be prognosticated and included in a counseling session about treatment options for DDH.

## Background

Developmental dysplasia of the hip (DDH) is an important cause of hip pain in young adults, often affecting them in the pursuit of high-level performances in sports, leisure activities, employment, and parenthood [1–4]. Understandably, affected patients demand information concerning the time course for symptom progression and on possible therapeutic options.

The relevant anatomical characteristic of DDH is a deficient acetabular development, leading to an acetabular under coverage of the femoral head [5, 6]. The sequelae of this morbid anatomy include axial overloading with decreased contact area, increased contact stress on the cartilage matrix with failure of the acetabular labrum, hypertrophy of the labral cartilage, maximum loading at the acetabular rim, and progressive instability, all of which accelerating joint degeneration [1, 2, 7]. Therefore, persistence of DDH beyond skeletal maturity predisposes to premature osteoarthritis of the hip and consequently to undergoing hip arthroplasty at a considerably young age [1, 6].

Initial symptoms of DDH, typically activity-related hip pain due to muscular or articular overload and progressive instability, may be experienced for several years before OA evolves [2, 3]. The dysplastic fit of a non-osteoarthritic hip joint may be corrected via several non-arthroplasty procedures such as proximal femoral and periacetabular osteotomies [2, 8–11]. Among those, the Bernese periacetabular osteotomy (PAO) has been proven to be an effective technique to substantially delay or ideally prevent total hip arthroplasty (THA) in critically selected patients with closed triradiate cartilage and symptomatic dysplasia of the acetabulum [4, 12–14].

Most commonly, treatment decisions rely upon plain radiographs which are diagnostic gold standards for both DDH in adulthood and osteoarthritis (OA) [5].

A variety of radiographic features trying to quantify the coverage of the femoral head, and thus measuring the extent of acetabular dysplasia, have been described [5]. However, the predictive relevance of these radiographic parameters concerning the progression of degenerative changes, and consequently the time frame for a possible THA, has mostly remained unclear. This study will analyze the connection between individual radiographic hip joint geometry and timing of primary THA, with the goal to devise a mathematical and graphical correlation enabling an estimation of the approximate patient age at the time of replacement of a dysplastic hip joint.

## Materials and methods

All patients who underwent total hip arthroplasty (THA) for osteoarthritis secondary to developmental dysplasia of the hip (DDH), between 2008 and 2014, at our institution, were considered for inclusion in this retrospective study. The indication for THA has always been consistent with radiological, anamnestic, and clinical criteria. The radiologic indication required osteoarthritis (OA) grade ≥ 3 according to Kellgren and Lawrence [15]. Anamnestic indication were significant restrictions in everyday life and leisure time, restrictions of walking distance less than 200 m, daily or regular use of analgesics, and pain at rest or at night. In the clinical examination, painful movement restrictions, positive pain in the groin, buttock, and greater trochanter, limping, and painful gait pattern had to be present.

Prerequisite for inclusion into our analyses was an acetabular dysplasia defined as an LCE angle ≤ 30° and/or acetabular index (AI) ≥ 10°. In addition, a series of X-ray images showing the course of osteoarthritis had to be available for each patient so that the radiological measurements could be performed on nearly healthy hips without osteoarthritis-related changes (osteophytes) ensuring correct angle measurements. To scrutinize the exclusive impact of acetabular configuration, inclusion was further limited to patients with a spherical femoral head and congruent acetabular fit (type 1 and 2 according to Stuhlberg’s classification). An LCE angle > 30°, an AI < 10°, high dislocation of the hip (type 4 according to Crowe’s classification), and/or a history of previous hip surgery during adulthood (e.g., femoral or acetabular corrective osteotomies) led to exclusion from this study.

**Fig. 1:**
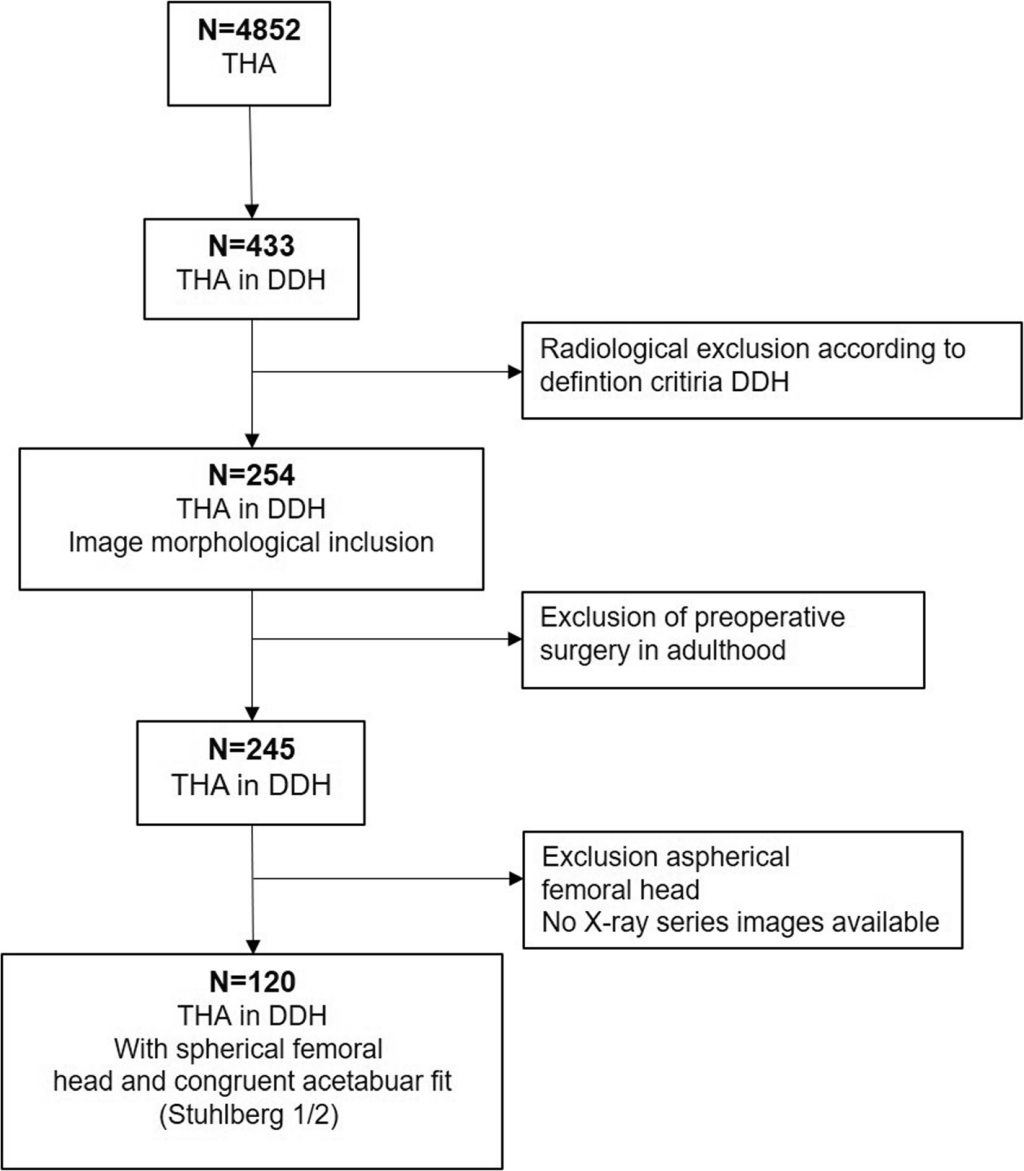
Flow chart depicting patient selection. Patient selection was performed using the hospital information system (HIS). The procedure search after ICD-10 (5-.820**) (cemented or non-cemented THA) in the abovementioned period identified 4852 patients in total. According to the expert panel evaluation of their standard preoperative nonarthritic X-rays, 129 hips of 120 patients could be selected

Patient selection was performed using the hospital information system (HIS). The procedure search after ICD-10 (5-.820**) (cemented or non-cemented THA) in the abovementioned period identified 4852 patients. Four hundred thirty-three of these had been labeled as having undergone THA due to unilateral or bilateral osteoarthritis secondary to DDH and were therefore considered for inclusion. According to the expert panel evaluation, standard preoperative nonarthritic X-rays of 129 hips of 120 patients met all enrollment criteria (Fig. 1: flow chart depicting patient recruitment).

Two orthopedic (M.M.; G.W.) surgeons with profound expertise in diagnostics of hip joint pathologies independently performed arthro-geometry measurements using standard preoperative radiographs, including anteroposterior (AP) pelvic view centered over the hips in standing position to evaluate the acetabulum, pelvic slope, and joint space and a Lowenstein or table down lateral view.

The included patients’ baseline demographics were collected in a database. Preoperative joint parameters were then correlated with the patient’s age at primary THA (pTHA).

Patients’ preoperative plain radiographs were evaluated using Ge-Healthcare software (Centricity™ Universal Viewer). All lengths, angles, and distances were measured to scale.

**Fig. 2:**
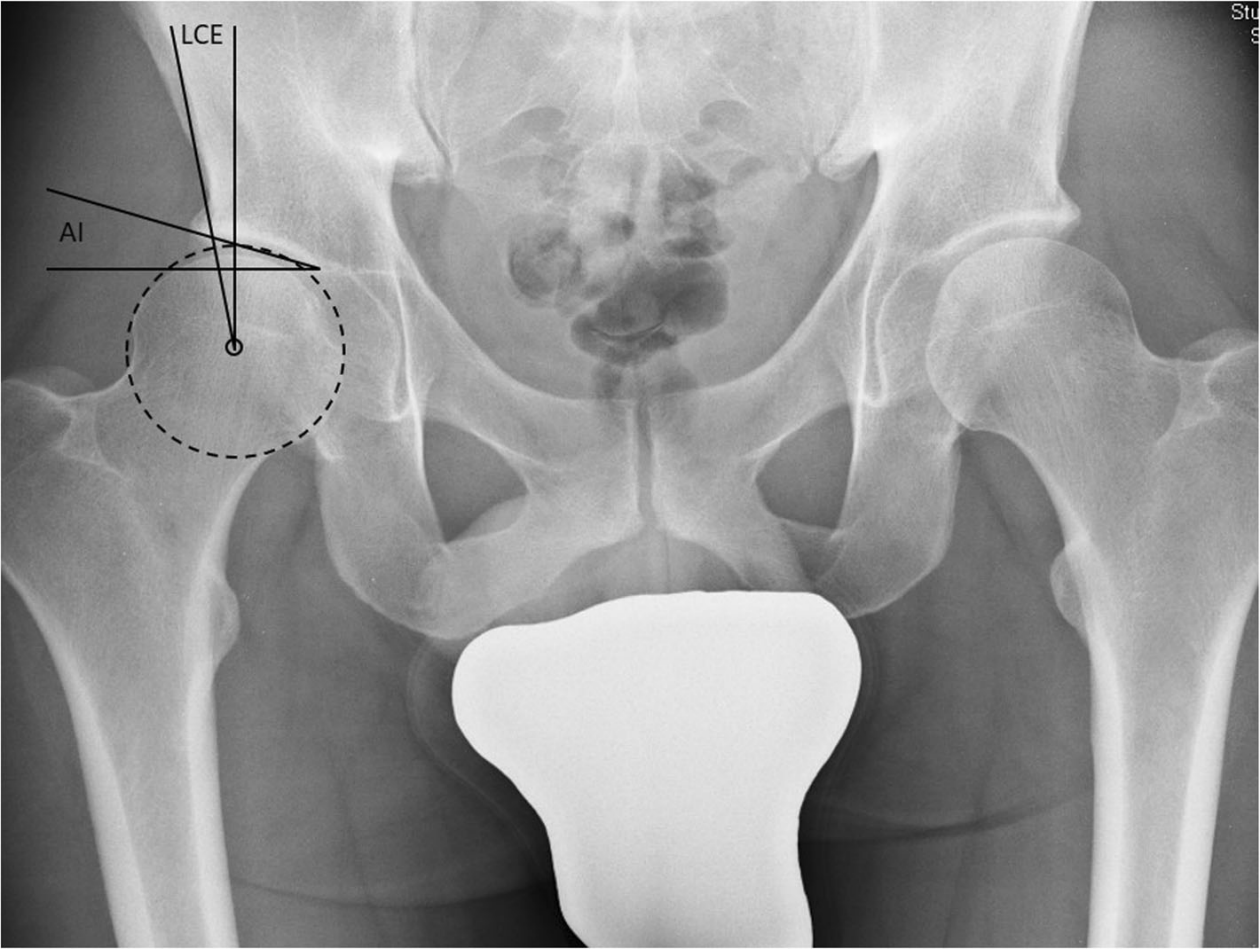
Exemplary radiograph (pelvic view) which shows the measurement of the LCE (Wiberg’s lateral center-edge) and AI (Hilgenreiner’s articular cartilage angle)

Radiographic arthro-geometry measurements included Wiberg’s center-edge (LCE) angle, AI (Hilgenreiner’s articular cartilage angle) (Fig. 2), Stuhlberg’s classification, and Crowe’s classification. The severity of radiographic OA was graded using the Kellgren and Lawrence classification. LCE angle and AI were chosen because these angles represent the most relevant radiographic parameters to assess the severity of dysplasia.

Additionally, the patients were divided into three groups to enable a clearer and clinically more useful evaluation: those with a CE angle < 10°, with a CE angle between 10 and 20°, and those with a CE angle of > 20°. Regarding the AI, the patients were divided into patients with an AI > 31°, between 21 and 30°, and between 10 and 20°.

### Statistics

Statistics were performed using SPSS (22.0, IBM, New York, USA). To measure the linear dependence (correlation) between the LCE, AI, and age at pTHA, Pearson’s correlation analyses were performed and positive or negative effects were deducted. The Spearman’s correlation coefficient was determined for variables that were not normally or ordinally distributed. Besides, the body mass index was correlated to age at pTHA according to a Pearson’s analysis. Via linear regression analysis with retrospective selection, a model-based formula for calculation of the anticipated age at pTHA was developed.

In order to further assess the relevance of LCE angles and AI, i.e., the severity of acetabular dysplasia, patients were grouped according to these parameters (LCE ≤ 10°, 11–20°, 21–30°, AI 10–20°, 21–30°, > 30°) and the groups were compared to their mean age at pTHA.

To start with, the entire model was tested for a significant age difference between the groups using ANOVA (analysis of variance). If groups differed significantly regarding age at pTHA, homoscedasticity (homogeneity of variance) was tested via Levene test and a post hoc test was chosen accordingly. With respect to the prevalent inequality of group size, the Scheffé test (very conservative) was applied. In case of equality of variance the least significant difference test (LSD test) (liberal) was deployed. For not normally distributed variables, the Kruskal-Wallis-test or the Mann-Whitney test was used.

Significance was set at *p* = 0.05. Adjustment for multiple tests was not performed.

To derive an equation for calculating of the probable patients’ THA age, as a function of joint parameters (LCE angle and AI), the method of power least squares curve fitting with second-degree polynomials were used (Mathcad express, MCG-Service GmbH, 83209 Prien am Chiemsee, Germany).

## Results

### Patient demographics

The mean patient age at the time of primary THA was 54.3 ± 11.3 years (range 15–78 years). Females outnumbered males significantly by a factor of 4.5. Table 1 summarizes the study population’s baseline data.

**Table 1:** Patient demographics

Total study population (hips/patients)	129/120
Females (hips/patients)	111/105
Males (hips/patients)	18/15
Right hips/left hips	73/56
BMI [kg/m^2^]	26.5 ± 5.9
Mean age at primary THA (years)	54.3 ± 11.3

**Fig. 3:**
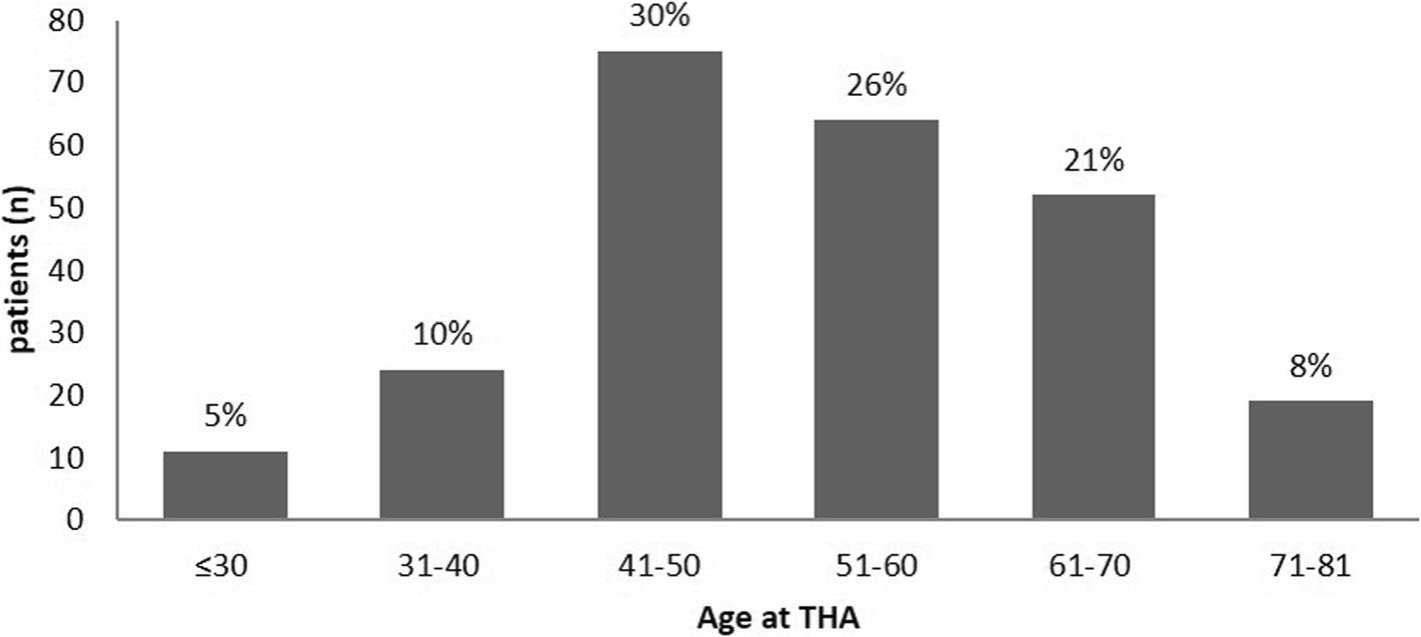
Age distribution at the time of primary THA. The study population’s mean age at index surgery was 52.9 ± 12.2 years. The majority of patients underwent pTHA in their fifth or sixth decade (56%)

Seventy-one percent of the patients were under 61 years of age, 45% were younger than 51 years, and 15% were younger than 41 years when undergoing primary THA. Figure 3 Depicts the distribution of age at time of primary THA among the study population

Most of the patients had been diagnosed with DDH early after birth (*n* = 50, 42%) or after the 18th year of life in the adulthood (*n* = 52, 43%). The rest of the patients’ DDH was diagnosed during childhood (*n* = 18, 15%).

Twenty-five percent of the study population (*n* = 33) had undergone corrective femoral or pelvic osteotomies during childhood or adolescence, 19% (*n* = 25) had a history of Pavlik harness therapy, and 55% (*n* = 74) had not undergone any therapeutic intervention previously.

Mean age at THA was 46.9 ± 12.3 years in the group of patients with previously surgically addressed hip joints, 53.4 ± 8.1 years in patients with previously merely conservatively treated DDH, and 58.2 ± 11.3 years in the group without previous DDH therapy.

The LSD test revealed statistically significant differences concerning age of patients at the time of primary THA between conservatively and surgically pre-treated DDH (*p* = 0.029) as well as between patients with a history of previous ipsilateral surgery during childhood and those who had not undergone any therapy (*p* ≤ 0.001). Approximately 60% of all previously surgically addressed hip joints had a higher degree of acetabular dysplasia (CE < 20°).

Patients included in this study reported to have suffered from permanent pain for a mean period of 12.2 ± 15.1 years prior to pTHA. Mean pain intensity during that period was 5.6 ± 2.8 on an 11-point numeric scale (NRS 11) with 0 representing “no pain” and 10 representing “worst pain imaginable”.

### Evaluation of radiographs of the hip joint

The mean LCE angle of the whole patient’s cohort was 21.2° ± 7.1° (range 0–30°). The mean AI was 23.2° ± 6.3° (range 10–48°). The mean Crowe type was 2 (range 1–3). The interobserver reliability was good with a kappa of 0.84.

### Age at primary THA and hip joint geometry

To better assess the link between severity of acetabular dysplasia and the age at primary THA, patients were grouped into three arrays according to the LCE angle and into three arrays according to the AI. The LCE angle arrays were LCE ≤ 10°, LCE 11–20°, and LCE 21–30°. Table 2 summarizes the distribution of age at pTHA according to LCE angle array. Unifactorial variance analysis revealed statistical significance of the difference of age at pTHA between these groups for the entire model (*p* ≤ 0.001). The Levene test demonstrated equality of variance (*p* = 0.592). The consequently as a post hoc analysis applied Scheffé test showed a statistically significant difference of age at pTHA between the ≤ 10° array and the 11–20° array, as well as between the ≤ 10° array and the 21–30° array (*p* ≤ 0.01).

**Table 2:** Age distribution at pTHA according to the LCE angle. Unifactorial variance analysis revealed statistical significance concerning the difference of age at pTHA between these groups for the entire model (*p* ≤ 0.001). On average, patients with an LCE angle ≤ 10° required THA more than 10 years earlier

LCE angle	≤ 10°	11–20°	21–30°
Number of patients (*n*)	12	37	80
Mean age at THA (years)	41.9 ± 14.0	52.7 ± 9.5	57.0 ± 10.3
Range of age at THA (years)	[15 to 68]	[33 to 73]	[30 to 78]

The LSD test additionally demonstrated a significant difference between patients with LCE angles of 11–20° and of 21–30°.

The AI arrays were > 31°, 21–30°, and 10–20°. Table 3 summarizes the distribution of age at pTHA according to AI array. Unifactorial variance analysis showed statistical significance of the difference of age at pTHA between these groups for the entire model (*p* ≤ 0.001). The Levene test demonstrated equality of variance (*p* = 0.699). The Scheffé test revealed a statistically significant difference of age at pTHA between the AI 21–30° array and the AI 31–60° array (*p* ≤ 0.05).

**Table 3:** Age at primary THA according to AI arrays. Statistically significant age differences between these groups were detected (*p* ≤ 0.01). The one-factor analysis of variance showed a significant difference in age between the individual groups (*p* ≤ 0.001). On average, patients with an AI > 31° required THA more than 10 years earlier

Acetabular index	10–20°	21–30°	> 31°
Number of patients (*n*)	50	63	16
Mean age at THA (years)	57.9 ± 10.0	53.7 ± 11.1	45.8 ± 11.3
Range of age at THA (years)	[30 to 76]	[20 to 78]	[15 to 68]

The Mann-Whitney test further demonstrated a significant difference between patients with AI of 10–20° and 21–30°(*p* ≤ 0.05) and for patients with AI of 10–20° versus AI of 31–60° (*p* ≤ 0.001).

**Fig. 4:**
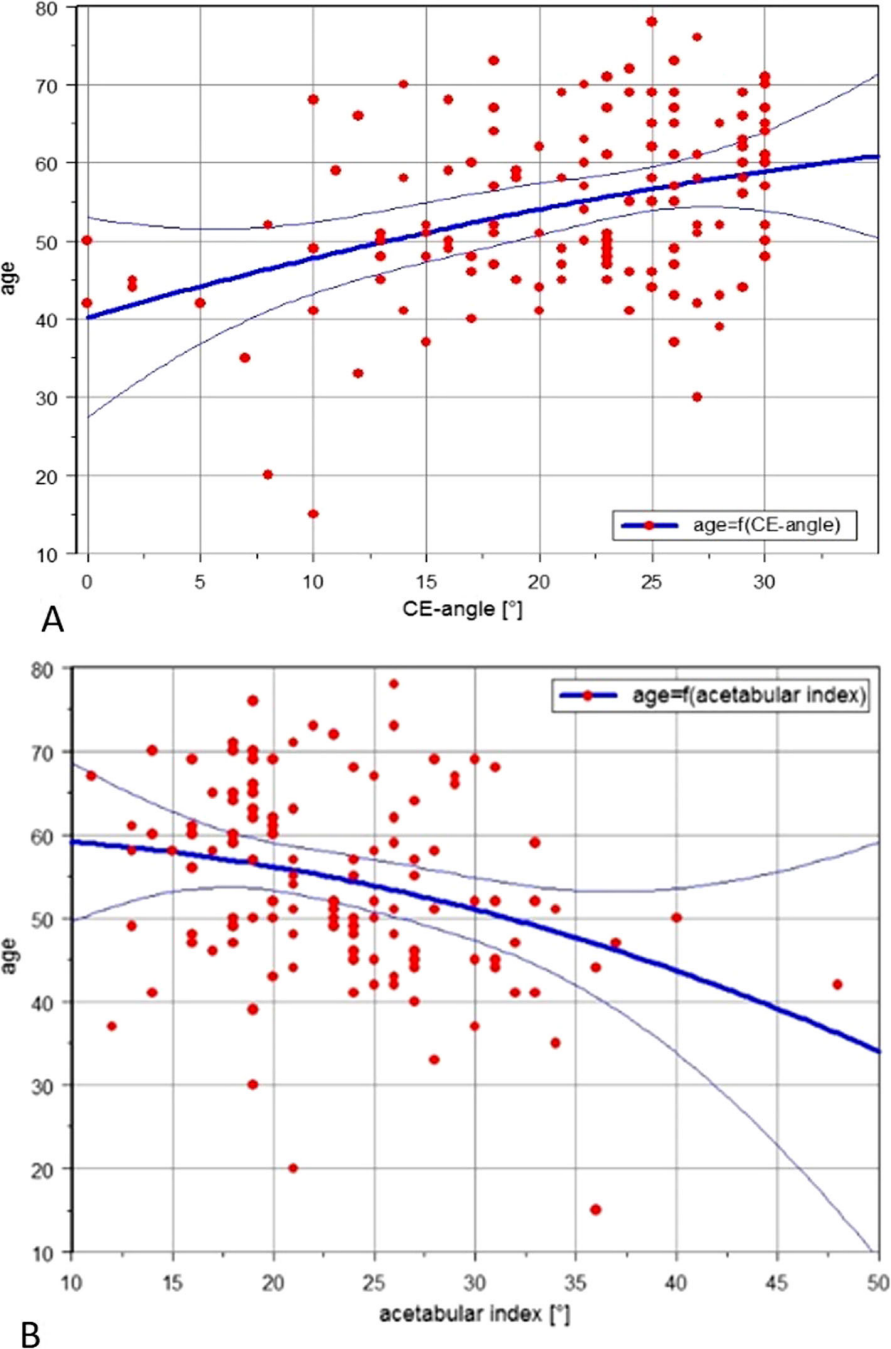
Scatter plots illustrating the statistical relationship of age at pTHA, LCE angle (**a**) and AI (**b**). *R* is 0.37 and − 0.29, respectively. These scatter plots of the LCE and acetabular indices against age illustrate the unambiguous relationship of age in primary THA with LCE angle and AI. Thus, the mean possible age of the necessary prosthesis implantation as a function of LCE angle and AI can be read from these diagrams

Figure 4 depicts the linear dependence of age at pTHA on LCE angle and AI. These scatter plots of the LCE and acetabular indices against age illustrate the unambiguous relationship of age in primary THA with LCE angle and AI. Thus, the mean possible age of the necessary prosthesis implantation as a function of LCE angle and AI can be read from these diagrams.

The LCE angle was positively correlated with the age of prosthetic implantation (*R* = 0.37), while the AI correlated negatively (*R* = − 0.3).

Neither correlation analysis nor linear regression analysis identified a significant correlation between the body mass index (BMI) and age at pTHA (correlation coefficient is 0.014; regression coefficient is 0.028; both non-significant).

Bivariate regression analyses revealed that LCE angles and AI (*p* ≤ 0.001) had significant impact on the age at pTHA.

A multivariate regression analysis allowed for the determination of the probable age at primary (p)THA. Age at pTHA was the dependent variable. The LCE angle was identified to be the radiographic parameter with the highest significant impact, which allowed for deduction of the formula to predict age at pTHA. According to the distribution of the value pairs in the scatterplot, the following formula has been calculated as the result of the method of power least squares curve fitting with second-degree polynomials to calculate the age at pTHA as a function of the LCE angle.

Age at pTHA f[LCE angle] = 40.2 + 0.8 × LCE angle − 0.01 × (LCE angle)^2^

Table 4 lists regression coefficient, standard error, *t* value, and *p* value (*n* = 129) of LCE angle and of the constant calculated by multivariate regression analysis (*n* = 129).

**Table 4:** Depiction of regression coefficient, standard error, *t* value and *p* value of LCE angle and of the constant calculated by multivariate regression analysis. Multivariate regression analysis allowed for determination of the probable age at primary (p)THA. Age at pTHA was the dependent variable. The LCE angle was identified to be the radiographic parameter with the impact of highest significance

Independent variable	Regression coefficient	Standard error	*t*	*p*
LCE angle (°)	0.583	0.131	4.435	0.000
Constant	41.985	2.935	14.307	0.000

The adjusted *R*^2^ amounts to 0.127.

## Discussion

One of the main goals of the study was to devise a mathematical and graphical characterization estimating the patient age at the time of replacement of a dysplastic hip joint as a function of the hip joint geometry. With this present investigation, we were able to calculate the relationship between the severity of the dysplasia and the associated time of the necessary prosthesis implantation.

The median age of the study group at the time of primary THA was 54 years. As expected, this was much younger than in patients with primary arthritis of the hip, in which the mean pTHA age is rather between 65 and 70 years [15, 16]. Crowe et al. examined 31 patients with THA due to secondary osteoarthritis caused by DDH. In their patient collective, the mean age was 57 years at the time of surgery [17]. In the study of Roidis et al., who also investigated patients with THA following DDH, the mean age of the patients at the time of surgery was 43 years (23–55), but 40% of the collective had a high hip dislocation [16]. Unfortunately, none of these studies provided a correlation between the anatomical characteristic of the dysplasia and the time of implantation.

In total, 4.5 times more female patients were treated in this study, which also corresponds to the latest literature results [18].

The anatomical joint parameters of the study collective were in the dysplasia range and also corresponded to the data from the literature [5, 6].

One of the main goals of this study was to investigate the influence of dysplastic hip joint morphology on the timing of hip THA. The results show a clear association between the dysplastic hip anatomy and the age of prosthesis implantation. In particular, the LCE angle and the AI correlated significantly with the age at the time of THA. The more dysplastic the angles were, the sooner the hip prostheses implantation was necessary. For example, patients with an LCE angle of less than 10° were on average 10 years earlier in need of a THA than patients with an LCE angle between 10 and 20° and on average 15 years earlier compared to patients with an LCE angle between 20 and 30°. The situation is similar with regard to the AI. Likewise, patients with an AI greater than 30° had a THA approximately 8 years earlier than patients with an AI between 20 and 30° and 12 years earlier compared to patients with an AI between 10 and 20°. Patients with an LCE angle of less than 10° and an AI of more than 30° underwent THA on average in the fifth decade of life or the early 40’s. In contrast, patients with LCE angles between 20 and 30 ° or an AI in between 10 and 20° had their THA on average only in the sixth decade of life, at the end of their 50’s.

The mathematical and graphical relationship between the severity of the dysplasia and the influence on the time of prosthesis implantation can be used as an advisory tool to give patients with DDH an orientation as to when the implantation of a prosthesis might become necessary. Additionally, the graph or formula may be helpful when considering the indication for a corrective pelvic osteotomy, for example, a young female presents in the outpatient clinic with symptomatic DDH and an LCE angle of approx. 7° without signs of hip arthritis. Following the results of this study, it now could be presumed that without a corrective osteotomy, the implantation of a prosthesis might be necessary between the 40th and 45th years of life.

To our knowledge, this is the first study examining the age dependence of prosthetic implantation on the relevant joint parameters, LCE and AI. Previous studies only demonstrated that patients with dysplastic hip joint have a significantly higher risk of premature osteoarthritis and therefore of early need of prosthesis implantation. Thus, Murphy et al. demonstrated that there was a clear dysplastic articular anatomy in patients with prosthetic implantation less than 65 years of age compared to those who later developed osteoarthritis [1]. In the group < 65 years, the mean LCE angle was 7 ± 12° [− 22° − 28°], compared to the group without prosthesis implantation older than 65 years in which the LCE angle was 34 ± 9°. Similarly, the AI was 25 ± 10° in the group older than 65 years, compared to the premature THA in the age group of < 65 years, which had an LCE angle of 6 ± 6° [1]. A further study by Wyles et al. demonstrated that patients with dysplastic hip morphology (LCE angle < 25 °) and onset of initial degenerative changes (Tönnis 1) were at higher risk of rapid progression of osteoarthritis within the next 10 to 20 years as compared to patients without dysplasia and initial osteoarthritis. Thus, it emerges that the probability of THA in patients with DDH and initial degenerative changes within the next 10 years are at one to three or two to three after 20 years [6].

The average age of THA in patients with DDH is well below that of patients with non-dysplastic osteoarthritis. In this context, it should also be mentioned that on average, preoperative pain averaged 12 years before THA and was approximately 5.6 on the VAS. Accordingly, the pain occurred on average at the age of 40.7 years related to the entire patient population. Thus, painful restrictions in recreational and everyday activities have been present before the necessary prosthesis implantation and also in a very active phase of life. In a study by Hartofilakidis et al., the mean age of pain onset of patients with DDH was at the age of 34.5 years. With additional subluxation of the hip, this was even at 32.5 years, and in the presence of a high hip dislocation at about 31 years [17].

The limitations of this study include the retrospective design and the missing control group. Only the data of patients who received a prosthesis because of their DDH coxarthrosis were considered, but not those of patients with dysplastic joint anatomy, in which the implantation of a prosthesis was not necessary, or the expression of a terminal arthritis of the joint was present. On the one hand, this would affect the graphics and, on the other hand, further statements about the probability of prosthesis implantation would be possible. Thus, based on the data presented, a statement about the possible age of THA implantation can be made, but not with what probability the implantation of a prosthesis will be necessary. A further aspect to discuss is the inclusion of patients with an LCE angle < 30°, given the fact that in some studies dysplasia is only defined as an LCE angle < 25°. While this more limited LCE value may increase the accuracy of the formula and the graph, the inclusion of patients with borderline dysplasia better reflects the reality in clinical practice. Furthermore, we only included patients with a spherical femoral head and congruent acetabular fit to scrutinize the exclusive impact of acetabular configuration only. A non-spherical head or a non-congruent acetabulum would additionally influence the development of an arthritis of the hip and with that the time of THA.

## Conclusion

To give a resume, a clear mathematical correlation between the manifestation of hip dysplasia and the influence on the time of prosthesis implantation could be demonstrated. At an LCE angle below 10° and an AI angle > 30°, the age of THA was well before the 50th year of life. The results presented can be consulted as a decision guidance for patients with symptomatic DDH regarding the necessity of a pelvic osteotomy. Further investigations with a larger number of patients and the consideration of an untreated control group would be necessary to improve accuracy and to be able to make statements on the probability of THA.

## Data Availability

The datasets used and analyzed during the current study are available from the corresponding author on reasonable request.

## Abbreviations

AI: Acetabular index
DDH: Developmental dysplasia of the hip
LCE: Wiberg’s lateral center-edge angle
OA: Osteoarthritis
THA: Total hip arthroplasty
